# Association of Self-esteem with Mental Health and Personality: The Contribution of Genetic and Environmental Factors

**DOI:** 10.1007/s10519-025-10249-7

**Published:** 2026-01-07

**Authors:** Henrike F. van den Berg, Meike Bartels, Bruno Sauce, Teemu Palviainen, Richard J. Rose, Jaakko Kaprio, Eero Vuoksimaa, Karri Silventoinen

**Affiliations:** 1 Department of Biological Psychology, Faculty of Behavioural and Movement Sciences, Vrije Universiteit Amsterdam, Amsterdam, The Netherlands; 2 Amsterdam Public Health Research Institute, Amsterdam University Medical Centres, Amsterdam, The Netherlands; 3 Institute for Molecular Medicine Finland (FIMM), HiLIFE, University of Helsinki, Helsinki, Finland; 4 Department of Psychological & Brain Sciences, Indiana University, Bloomington, IN USA; 5 Helsinki Institute for Demography and Population Health, University of Helsinki, P.O. Box 42, Unioninkatu 33, FIN-00014 Helsinki, Finland

**Keywords:** Self-esteem, Mental health, Personality, Twins, Polygenic score

## Abstract

Individuals with high self-esteem experience less social anxiety, fewer depressive symptoms, and exhibit lower neuroticism and higher extraversion. We aimed to explore the genetic and environmental influences behind these associations. Self-esteem, four mental health indicators, and five personality factors were assessed in 1,288 Finnish young adult twins, including 583 complete pairs. The mean age of the participants was 21.9 years (SD = 0.8). Classical twin modelling was used to estimate genetic and environmental correlations. Additionally, regression models were used to examine the association between self-esteem and polygenic scores (PGS) of several mental health traits. Among all participants, self-esteem associated positively with extraversion, agreeableness, and conscientiousness and negatively with depressive symptoms, alexithymia, schizotypal personality, overall mental health problems and neuroticism. These associations were explained by additive genetic factors (19–66% of covariation) and unique environmental factors (36–81% of covariation) when using twin modelling. Self-esteem correlated only with the PGS of subjective well-being in men and women. The proportion of variance of self-esteem explained by the PGSs was minor (1.5% or less). These findings suggest that while self-esteem shares a genetic background with mental health and personality traits, unique environmental factors can also influence these connections. Our findings are consistent with a hypothesis that enhancing self-esteem can have a positive impact on mental health.

**Supplementary Information:**

The online version contains supplementary material available at 10.1007/s10519-025-10249-7.

## Introduction

Self-esteem, among other aspects of positive mental health, has been less studied than psychiatric disorders and psychopathology. Self-esteem refers to an individual’s subjective evaluation of their own worth and abilities, which determines whether they view themselves positively or negatively (Donnellan et al. 2011). High self-esteem has long been associated with happiness and life satisfaction (Diener and Diener 1995), and it is linked positively with work-related outcomes, such as job satisfaction, productivity, and feelings of fairness at work, as well as fulfilling relationships (Kuster et al. 2013; Orth et al. 2012). High self-esteem also serves as a protective factor against mental health problems during adolescence (Liu et al. 2021). Conversely, low self-esteem consistently co-occurs with mental health issues, such as social anxiety and depressive symptoms (Isomaa et al. 2013; Schreiber et al. 2012; Sowislo et al. 2014). High self-esteem is also positively correlated with extraversion, conscientiousness, agreeableness, and openness and negatively correlated with neuroticism when measured using the Big Five personality scale (Robins et al. 2001; Shikishima et al. 2018).

Several factors can influence the co-occurrence of low self-esteem and mental health problems. Psychosocial factors, such as childhood difficulties (Volanen 2004) and insecure attachments as infants (Malekpour 2007), can lead to low self-esteem and have a negative impact on mental health. However, a shared genetic background can also explain the associations between self-esteem and mental health. Twin studies have shown that around 30–60% of the variation in self-esteem is attributed to genetic differences (Raevuori et al. 2007; Roy et al. 1995; Stieger et al. 2017). Because mental health problems, including depression and anxiety (Hettema et al. 2001; Sullivan et al. 2000), are also heritable, they could be influenced by genetic factors overlapping with the genetic factors affecting self-esteem. Although this has not been studied for mental health, a Japanese twin study of 520 pairs indicated genetic overlap between self-esteem and personality factors (Shikishima et al. 2018).

We analysed the relationship between self-esteem, mental health, and personality to understand how genetic and environmental factors contribute to these associations. We used a twin model to estimate genetic and environmental influences on the covariance and calculate genetic and environmental correlations between these psychological traits. Additionally, we examined how polygenic scores (PGS) of various mental health and personality indicators were associated with self-esteem. These two methods make different assumptions and complement each other when used together (Friedman et al. 2021). The Hierarchical Taxonomy of Psychopathology (HiTOP) model suggests a general factor of psychopathology (Conway et al. 2022), and previous research shows that this factor can correlate with a broad personality factor (McCabe et al. 2022). This is supported by a recent study showing broad genetic overlap between different psychiatric disorders (Grotzinger et al. 2025). Therefore, we selected various mental health indicators and PGS along with personality factors to study how self-esteem fits into this general framework.

Based on previous research, we expected significant associations between self-esteem, mental health, and personality (Isomaa et al. 2013; Shikishima et al. 2018). Our genetically informative data can provide insights into the underlying factors of these associations, which could have implications for interventions. If genetic factors, potentially reflecting a common neurophysiological background, explain these associations, we expect to see only genetic correlations using both study designs. If the association is driven by individual-level experiences, we would expect to see correlations for environmental factors specific to each twin individual. A possible causal association between self-esteem and mental health indicators would increase both additive genetic and unique environmental correlations.

## Data and method

**Sample**

We utilized data from the FinnTwin12 study, in which the target population was all twins born in Finland between 1983 and 1987 identified through the Finnish Central Population Registry (Kaprio et al. 2002). All twin pairs with both twins alive and residing in Finland were invited to participate in the study at the age 11/12 baseline. The relevant data for our analyses were collected during the fourth wave when the twins were 20–27 years old (mean age 21.9, standard deviation (*SD*) = 0.8). Selected participants underwent an in-person intensive study where they completed questionnaires on self-esteem, mental health, and personality. Out of the 1852 invited twins, 1290 returned the questionnaire yielding a response rate of 70%. Most twins also provided a blood sample for DNA extraction. Zygosity was determined by genotyping information or by using questions about physical similarities in the baseline questionnaire when genotypic information was not available. This questionnaire method has demonstrated high reliability in this dataset (Jelenkovic et al. 2011). Two individual twins without confirmed information on zygosity were excluded from the final sample, resulting in a sample size of 1288 twins (54% women). We had a total of 254 monozygotic (MZ), 176 same-sex dizygotic (DZ) and 156 opposite-sex DZ complete twin pairs.

**Measures**

Self-esteem was assessed using the 10-item Rosenberg Self-Esteem Scale, which is a unidimensional measure of global self-esteem that evaluates overall feelings of self-worth and self-acceptance (Rosenberg 1965). The total score ranges between 10 and 40, with higher scores indicating better self-esteem. Depressive symptoms were measured using the 10-item short version of the General Behavior Inventory questionnaire, which focuses on mood-related behaviors, such as depressive, hypomanic, and biphasic symptoms (Depue et al. 1981). This scale includes 10 items that assess the occurrence of depressive symptoms, with responses on a 4-point Likert scale ranging from 0 (*never*) to 3 (*very often*) resulting in a total sum score of 0–30 (Ranjit et al. 2019). Alexithymia was assessed with the 20-item Toronto Alexithymia Scale, which measures difficulties in describing and identifying emotions (Bagby et al. 1994). Responses were provided on a five-point Likert scale, and the total score ranges between 20 and 100, with a higher score indicating more prominent alexithymia. Schizotypal personality traits were evaluated using the 22-item Schizotypal Personality Questionnaire-Brief (O’Hare et al. 2020; Raine and Benishay 1995). Each affirmative response on the questionnaire received a score of 1, yielding a total score ranging between 0 and 22, with higher scores indicating a more schizotypal personality. Overall mental health problems were assessed with the Goldberg 12-item General Health Questionnaire (GHQ), which inquiries about general mood and mental health issues in daily life (Goldberg et al. 1970; Penninkilampi-Kerola et al. 2006). Responses were rated on a four-point Likert scale ranging from 0 (not at all) to 3 (much more than usual). A total score ranges between 0 and 36, with higher scores indicating greater distress. Personality traits were assessed using a Finnish version of the Big Five Personality Inventory (Pulver et al. 1995). This version is based on a shortened form of the original 180-item NEO Personality Inventory (NEO-PI) known as NEO-FFI (Costa Jr and McCrae 1989), which consists of 60 items. Additionally, seven extra items assessing sensation-seeking were included in the extraversion scale, resulting in a total of 67 items.

The Cronbach’s alpha coefficients for these scales ranged 0.7–0.9, indicating good internal consistency in the data used in this study (Silventoinen et al. 2022). Most personality and mental health traits showed good or satisfactory symmetry (the absolute value of skewness 0.05–0.96). However, depression and overall mental health displayed higher skewness (1.38–1.77) along with kurtosis (5.10–8.57) (Supplementary Table 1). Despite this, we chose not to statistically transform these variables because we were especially interested in their covariation with self-esteem, and such transformations may have introduced bias to the estimates.

**Data analysis**

To investigate the impact of genetic and environmental factors on the relationships of self-esteem with mental health and personality, we utilized genetic twin modelling (Posthuma et al. 2003). MZ twins are virtually identical at the gene sequence level while DZ twins share, on average, 50% of their genes identity-by-descent. By applying these assumptions to structural equation modelling, the trait variance and covariance between traits can be decomposed into additive genetic (A), shared environmental (C) or dominant genetic (D), and unique environmental (E) components. We have previously reported that genetic factors account for between 19% and 66% of the variation in these traits while the rest of the variation was explained by unique environmental variation (Silventoinen et al. 2022). No evidence was found for dominant genetic or shared environmental variation leading us to use the additive genetic/unique environment (AE) model. We identified a statistically significant sex-specific genetic factor for four traits and different sizes of variance components in men and women for six traits, as previously reported (Silventoinen et al. 2022). Consequently, we conducted separate analyses for each sex. Age effects were not found primarily due to the narrow age range of these young adult twins, and therefore we did not include age as a covariate. We employed the computationally robust bivariate Cholesky decomposition of the AE model, which calculates latent additive genetic and unshared environmental factors for the variation and co-variation between the traits (Kaprio and Silventoinen 2011). This method allowed us to determine the additive genetic and unique environmental correlations and estimate how much these factors explain the associations of self-esteem with mental health and personality. Using this method, we divided the variance of each trait into additive genetic and unique environmental variation shared with self-esteem and specific to the trait. We also tested the potential impact of having a same-sex or opposite-sex co-twin on self-esteem and the other psychological traits by comparing the means stratified by zygosity, as this has not been systematically analysed previously.

Additionally, we calculated the associations between self-esteem and PGS for major depressive disorder, schizophrenia, broad depression, neuroticism, subjective well-being, externalized problems, and mental health problems. We had genetic data available for 1,260 twin individuals. The PGS were calculated from the summary statistics of previously conducted genome-wide association studies (GWAS). The technical details of genotyping have been described elsewhere (Kujala et al. 2020). Calculation of p-values and 95% confidence intervals for PGS and descriptive statistics were done using regression models by Stata statistical package, version 17 for Windows, after correcting for the lack of statistical independence of twins sampled as pairs (Williams 2000). The OpenMX package version 3.0.2 of R version 4.2.2. was used for the twin modelling (Neale et al. 2016).

## Results

Table 1 presents the descriptive statistics for self-esteem and other psychological traits stratified by sex and zygosity. Significant differences were found between the zygosity categories for two traits when using the conventional significance level (*p* <.05). However, none of these differences were significant when using the Bonferroni corrected significance level (*p* <.0025 for 20 tests).

**Table 1:** Descriptive statistics of self-esteem, mental health, and personality, stratified by sex and zygosity

	MZ			Same-sex DZ			Opposite-sex DZ			*p*-value
	*N*	Mean	SD	*N*	Mean	SD	*N*	Mean	SD	
Men										
Self-esteem	236	33.8	4.80	180	32.9	5.01	170	32.6	4.97	0.0609
Mental health										
Depressive symptoms	236	13.5	4.21	181	13.7	3.95	170	13.8	4.16	0.8609
Alexithymia	236	29.0	9.81	182	30.0	9.00	168	30.3	9.30	0.4350
Schizotypal personality	231	4.9	4.33	175	5.2	4.17	164	5.4	4.40	0.6181
Overall mental health problems	236	21.0	4.43	179	21.3	3.84	168	21.7	4.38	0.2275
Personality										
Neuroticism	235	1.3	0.67	180	1.4	0.59	169	1.4	0.63	0.1352
Extraversion	235	2.5	0.46	180	2.4	0.45	169	2.4	0.43	0.3223
Openness	234	2.0	0.52	180	2.1	0.55	169	2.1	0.55	0.4359
Agreeableness	235	2.6	0.42	180	2.7	0.41	169	2.6	0.41	0.6938
Conscientiousness	235	2.6	0.54	180	2.5	0.51	169	2.4	0.55	0.0363
Women										
Self-esteem	300	30.6	5.81	203	29.8	5.46	194	30.9	4.96	0.1077
Mental health										
Depressive symptoms	300	14.9	4.76	204	16.1	5.70	192	15.0	4.25	0.0667
Alexithymia	300	26.9	10.18	204	29.2	10.03	194	27.6	9.99	0.1030
Schizotypal personality	294	6.3	4.57	196	6.5	4.55	186	5.4	3.94	0.0354
Overall mental health problems	300	23.3	5.45	204	24.2	6.22	193	23.2	4.76	0.2165
Personality										
Neuroticism	203	2.0	0.71	192	1.8	0.61	203	2.0	0.71	0.1204
Extraversion	203	2.3	0.39	192	2.3	0.39	203	2.3	0.39	0.2886
Openness	203	2.2	0.52	192	2.3	0.50	203	2.2	0.52	0.1578
Agreeableness	203	2.7	0.46	192	2.7	0.45	203	2.7	0.46	0.2174
Conscientiousness	195	2.5	0.53	152	2.6	0.53	195	2.5	0.53	0.5905

Table 2 presents the trait correlations of self-esteem with other psychological indicators, decomposition of these correlations into additive genetic and unique environmental correlations, and the proportion of trait correlations explained by additive genetic and unique environmental factors. When studying the associations with mental health problems, men and women with higher self-esteem had fewer depressive symptoms, less alexithymia, less schizotypal personality, and fewer overall mental health problems. No significant differences in the trait correlations were found between men and women (the p-values of sex interactions 0.34–0.79). The strongest association was found with schizotypal personality in men (*r* = −.55) and depressive symptoms in women (*r* = −.52). Both additive genetic (19% – 64%) and unique environmental factors (36% – 81%) explained a proportion of these trait correlations. When the trait correlations were decomposed, moderate to strong genetic (*r*_A_ = − 0.51 – − 0.88) and unique environmental correlations were found (*r*_E_=−0.36 – − 0.81).

**Table 2:** Trait, additive genetic and unique environmental correlations of self-esteem with mental health and personality factors and the proportions of the trait correlations explained by additive genetic and unique environmental factors in men and women.

	Trait correlation			Additive genetic factors				Unique environmental factors			
	*r*	95% confidence intervals		*r*_A_	95% confidence intervals		% explained	*r*_E_	95% confidence intervals		% explained
Men											
Mental health											
Depressive symptoms	–0.52	–0.57, –0.45		–0.54	–0.77, –0.23		38	–0.47	–0.60, –0.32		62
Alexithymia	–0.51	–0.57, –0.45		–0.88	–1.00, –0.62		47	–0.30	–0.44, –0.13		53
Schizotypal personality	–0.55	–0.61, –0.50		–0.74	–0.95, –0.54		52	–0.44	–0.57, –0.29		48
Overall mental health problems	–0.49	–0.55, –0.42		–0.58	–1.00, –0.58		19	–0.44	–0.56, –0.29		81
Personality											
Neuroticism	–0.70	–0.74, –0.65		–0.77	–0.92, –0.77		49	–0.62	–0.72, –0.50		51
Extraversion	0.46	0.39, 0.52		0.77	0.56, 1.00		57	0.23	0.06, 0.39		43
Openness	0.05	–0.04, 0.13		0.08	–0.16, 0.17		66	0.03	–0.15, 0.22		34
Agreeableness	0.29	0.21, 0.36		0.30	–0.24, 0.69		22	0.33	0.17, 0.48		78
Conscientiousness	0.32	0.24, 0.39		0.45	0.16, 0.73		59	0.15	–0.03, 0.31		41
Women											
Mental health											
Depressive symptoms	–0.52	–0.58, –0.47		–0.58	–0.68, –0.45		64	–0.48	–0.59, –0.35		36
Alexithymia	–0.46	–0.52, –0.40		–0.57	–0.71, –0.41		47	–0.34	–0.46, –0.20		53
Schizotypal personality	–0.48	–0.53, –0.42		–0.51	–0.63, –0.37		59	–0.45	–0.56, –0.32		41
Overall mental health problems	–0.51	–0.56, –0.45		–0.67	–0.81, –0.52		42	–0.36	–0.48, –0.22		58
Personality											
Neuroticism	–0.72	–0.75, –0.68		–0.78	–0.86, –0.69		59	–0.61	–0.70, –0.51		41
Extraversion	0.29	0.22, 0.36		0.30	0.13, 0.45		58	0.34	0.19, 0.46		42
Openness	0.12	0.05, 0.19		0.15	–0.02, 0.31		61	0.06	-0.09, 0.21		39
Agreeableness	0.19	0.12, 0.26		0.25	0.07, 0.41		57	0.06	–0.10, 0.21		43
Conscientiousness	0.27	0.20, 0.34		0.27	0.24, 0.45		53	0.17	0.01, 0.32		47

**Fig. 1:**
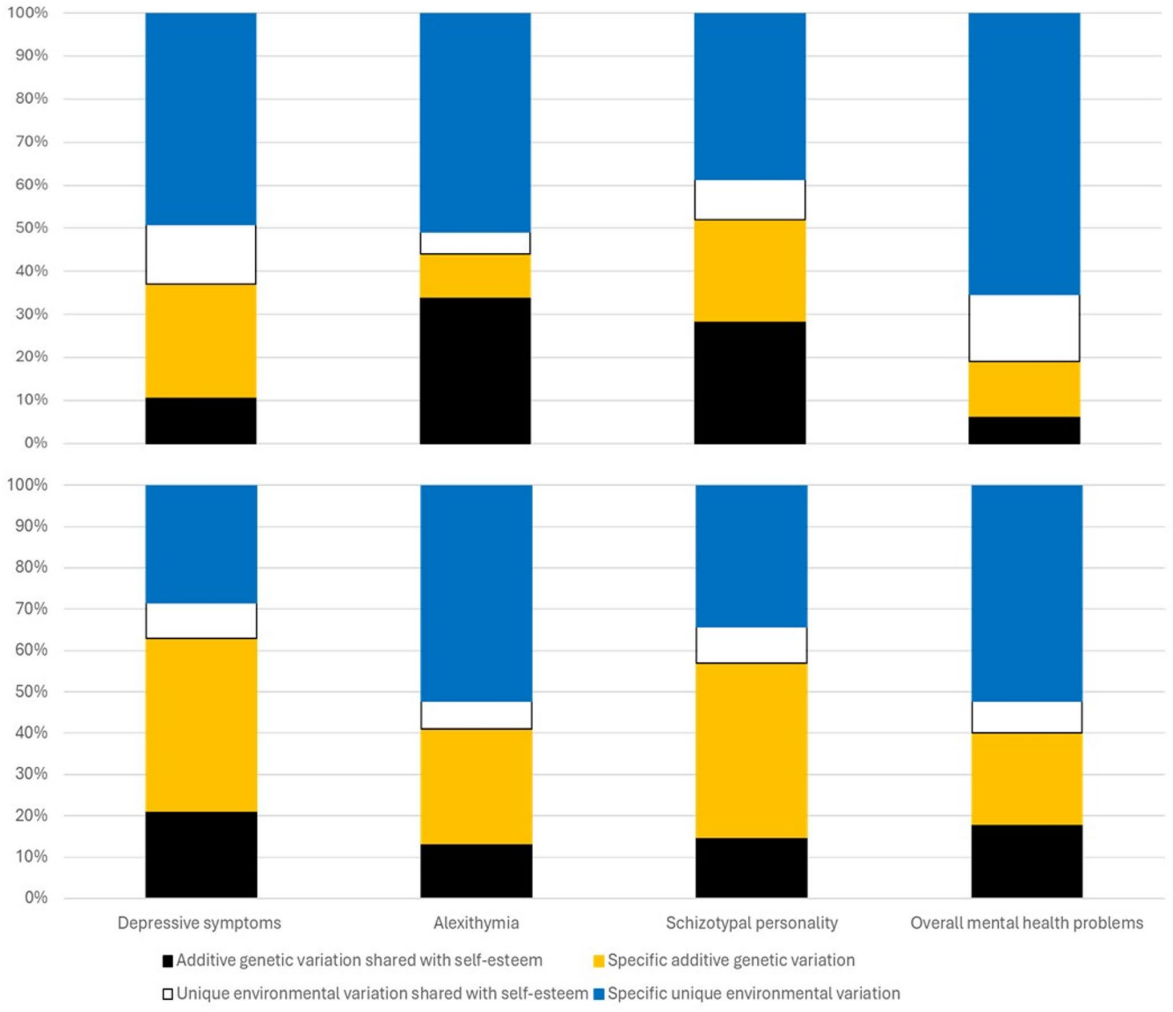
Decomposition of the variation in mental health indicators into additive genetic and unique environmental variation shared with self-esteem and specific to the trait in men (upper panel) and women (lower panel)

Figure 1 presents the variation of mental health indicators divided into additive genetic and unique environmental components shared with self-esteem and specific to each indicator. The proportion of the total variation shared with self-esteem varied between 20% and 49%. The proportion of the unique environmental variation shared with self-esteem varied between 5% and 16%. Sex differences in the size of variance components were statistically significant (*p* =.005 for schizotypal personality and *p* <.001 for other traits). The additive genetic variance shared with self-esteem was larger in men for alexithymia and schizotypal personality and in women for depressive symptoms and overall mental health problems.

We then analysed the associations between self-esteem and the five personality traits (Table 2). Self-esteem was negatively associated with neuroticism (*r* = −.70 for men and − 0.72 for women). In relation to the other personality traits, self-esteem showed positive associations, with the strongest correlation seen for extraversion (*r* =.46 and 0.29, respectively) and the weakest for openness (*r* =.05 and 0.12, respectively). There were no significant differences in trait correlations between men and women, with p-values for sex interactions ranging from 0.18 to 0.78. Additive genetic factors (22% – 66%) contributed to all these associations except for openness in men and women, and for agreeableness in men. Unique environmental factors (34% – 78%) contributed to all these associations except for openness in men and women, conscientiousness in men and agreeableness in women. When these trait correlations were decomposed, we found moderate to high additive genetic correlations (nominal values *r*_A_ = 0.25–0.77) and unique environmental correlations (nominal values *r*_E_ = 0.15–0.62).

**Fig. 2:**
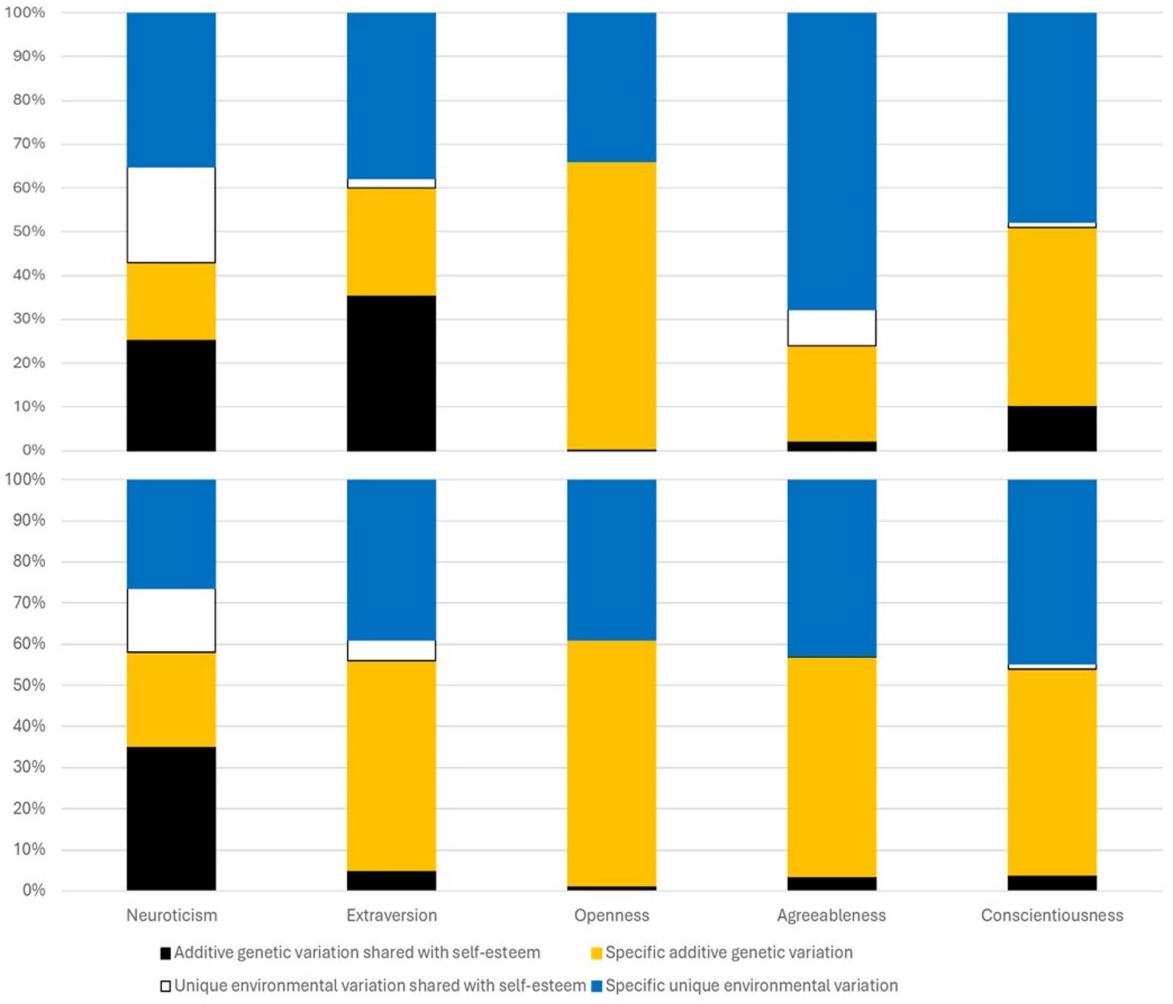
Decomposition of the variation in personality traits into additive genetic and unique environmental variation shared with self-esteem and specific to the trait in men (upper panel) and women (lower panel)

Figure 2 presents the decomposition of the variation of personality factors. Only neuroticism showed substantial covariation with self-esteem in both sexes (the proportion of total shared variation 47% in men and 51% in women; the proportion of shared unique environmental variation 22% and 26%, respectively). In men, extraversion also showed moderate shared total variation (38%), and the additive genetic variation shared with self-esteem was larger than that in women. The sex differences in the size of variance components were statistically significant (*p* =.05 for neuroticism, *p* =.006 for conscientiousness, *p* = .002 for openness, and *p* <.0001 for extraversion and agreeableness).

Finally, we estimated the correlations of the PGS from three mental health traits, neuroticism, and subjective well-being with self-esteem (Table 3). A higher PGS for well-being was associated with higher self-esteem in both men and women (*β* = 0.11 in men and 0.10 in women). The other correlations were not statistically significant. The PGS explained no more than 1.5% of the variance in self-esteem.

**Table 3:** Self-esteem regressed on polygenic scores of selected mental health measures in men and women.

	Men				Women			
	β	95% confidence intervals	*p*-value	% explained	β	95% confidence intervals	*p*-value	% explained
Major depressive disorder	−0.03	−0.11, 0.06	0.564	0.07	−0.07	−0.15, 0.01	0.071	0.56
Schizophrenia	0.03	−0.04, 0.11	0.382	0.13	−0.06	−0.16, 0.04	0.240	0.29
Broad depression	−0.02	−0.10, 0.06	0.673	0.04	−0.05	−0.13, 0.02	0.176	0.28
Neuroticism	−0.06	−0.15, 0.02	0.11	0.47	−0.09	−0.17, 0.00	0.039	0.76
Subjective well-being	0.11	0.03, 0.18	0.005	1.50	0.10	0.02, 0.19	0.041	1.16
Externalized problems	0.01	−0.06, 0.09	0.723	0.02	−0.04	−0.12, 0.04	0.317	0.19
Mental health problems	−0.05	−0.12, 0.02	0.199	0.30	−0.01	−0.09, 0.06	0.723	0.02

## Discussion

The current study provides evidence that self-esteem is associated with mental health and personality, consistent with findings from previous studies. The analyses using a twin design indicated that genetic and unique environmental factors both played a significant role in explaining these associations. We found little evidence that zygosity status or the sex of the co-twin affected self-esteem or other psychological traits. The negative correlations indicate that individuals with higher self-esteem experience fewer symptoms of depression, alexithymia, and schizotypal personality, as well as fewer overall mental health problems, aligning with previous research (Isomaa et al. 2013). Regarding personality traits, a strong negative correlation shows that individuals with higher self-esteem exhibit less neuroticism, and moderate correlations show that they also exhibit higher extraversion, agreeableness and conscientiousness. A weak correlation indicates that women with higher self-esteem also exhibit greater openness, but no significant association was found in men. The strength of the associations between self-esteem and personality factors was roughly similar to those calculated in a previous Japanese twin study of 151 twin pairs aged 14 to 30 years (Shikishima et al. 2018), showing that the associations are consistent in different cultural contexts.

Both genetic and unique environmental factors contribute to the associations of self-esteem with mental health and personality when using the twin design. The contribution of genetic factors is also supported by using PGS: the strongest association between self-esteem and PGS was found with the PGS of wellbeing. However, when using the PGS design, the proportion of explained variation in self-esteem was very low (1.5% or less) compared to the proportion explained by genetic factors using the twin design (an average of approximately 50%). This difference, known as missing heritability, is expected and commonly found in different traits (Young 2019). While twin studies estimate considerable heritability for several traits and diseases, the actual genetic factors in the genome identified with GWAS explain much less of the variation in the trait or disease than the twin estimate. This is likely due to a number of factors, including incomplete mapping and testing of genetic sites with GWAS, small GWAS sample sizes, and inconsistent classification of traits (Friedman et al. 2021). With whole-genome sequencing and larger sample sizes, the fraction accounted for by genetic factors increases substantially, even for behavioral traits such as smoking (Jang et al. 2022).

We found that the associations of self-esteem with mental health and personality factors were broadly similar in men and women. We have previously reported sex differences in the means of mental health and personality indicators in our data, with women showing lower self-esteem and more mental health problems. Differences were also found in the variance components of these indicators, warranting sex-stratified analyses (Silventoinen et al. 2022). This resulted in a different sharing of additive genetic and unique environmental variations between self-esteem and other psychological traits in men and women. Furthermore, women tend to exhibit more internalizing and men more externalizing mental health problems, which may be due to both biological and social factors (Christiansen et al. 2022). It is possible that despite the general similarity of the associations between self-esteem and other psychological traits in men and women, the background mechanisms may still differ.

The genetic correlations suggest that the genetic factors influencing self-esteem overlap with those affecting mental health and personality. One explanation for this genetic overlap is a shared biological basis for these traits. Positive (Alexander et al. 2021) and negative emotions (Arias et al. 2020) are known to have a neurobiological background, which may explain these genetic correlations. Additionally, unique environmental correlations may indicate that factors in one’s life experiences influencing self-esteem and mental health can also overlap. If these associations are solely due to a common neurophysiological background, we would expect unique environmental correlations to be significantly lower than additive genetic correlations.

Self-esteem is widely considered an important factor for mental health (Chen et al. 2025), and previous studies have proposed that self-esteem is a critical component in interventions aimed at promoting mental health (Mann 2004) and can act as a protective factor for mental health problems during adolescence (Triana et al. 2019). Our findings of genetic and unique environmental correlations between self-esteem and mental health symptoms are consistent with the hypothesis of a causal association, even though our cross-sectional data cannot confirm causality. From a clinical perspective, the most relevant result is the unique environmental component shared by self-esteem and mental health traits, as this represents a potential target for intervention. However, this component accounts for only a modest proportion of variance—approximately 5% to 16% across mental health indicators. Importantly, a causal association is only one possible explanation for the observed covariation. Within the HiTOP, a general factor of psychopathology has been proposed (Conway et al. 2022), which can further correlate with a broad personality factor (McCabe et al. 2022). In this framework, self-esteem could be interpreted as reflecting elements of a general personality factor, which might help explain its associations with mental health problems. Nonetheless, our results showed that self-esteem correlated moderately with mental health indicators and neuroticism but showed weaker associations with other personality dimensions. This pattern suggests that self-esteem may be more specifically tied to psychopathology rather than functioning as an indicator of general personality structure.

Our study has both strengths and weaknesses. Our main strength is the use of well-validated questionnaires to measure self-esteem, mental health, and personality in genetically informative data. This allowed us to examine the shared genetic background of self-esteem and other psychological traits using both the classical twin design, which utilizes the similarity of MZ and DZ twins, and the molecular genetic design, which analyses measured genetic polymorphisms. Each design uses different theoretical assumptions, and therefore the robustness of these results provides more convincing evidence of the shared genetic influences on these traits. A limitation of our study is that we relied solely on self-reported data, lacking information on clinically validated psychiatric diseases or ratings of personality factors by external sources. This reliance on self-reports may have introduced a response bias that could have strengthened trait correlations. The correlated measurement errors are likely to increase particularly unique environmental correlations. Therefore, our estimates should be considered as the upper limit of the unique environmental co-variation between self-esteem and mental health. It is also noteworthy that the indicators of mental health include items measuring feelings of self-worth, which overlap with the items of the self-esteem scale and thus increase correlations between self-esteem and mental health. However, this can also reflect real overlap between these concepts, as the lack of self-worth is an important part of depression. The indicators of depression and overall mental health problems also showed skewness, which may have affected the estimates of covariation with self-esteem.

In conclusion, the present study demonstrates the associations of self-esteem with mental health and personality traits. Both genetic and unique environmental factors specific to each twin played a role in these associations. Our study indicates that the link between self-esteem and other psychological traits is not solely a result of shared genetic background, but individual experiences and causality can also influence these connections. Our findings do not contradict the potential benefit of interventions aimed at enhancing self-esteem to promote better mental health.

## Supplementary Information

Below is the link to the electronic supplementary material.

Supplementary Material 1

## Data Availability

Because of the consent given by study participants and the high degree of identifiability, data cannot be made publicly available. Data are available through the Institute for Molecular Medicine Finland (FIMM) Data Access Committee (DAC) for authorized researchers who have IRB/ethics approval and an institutionally approved study plan. For more details, please contact the FIMM DAC (fimm‐dac@helsinki.f).
