# Supplementary material for: Association of Self-esteem with Mental Health and Personality: The Contribution of Genetic and Environmental Factors
Source: Behav Genet. 2026 Jan 7;56(1):29–38. doi: 10.1007/s10519-025-10249-7 (PMC12795968; doi:10.1007/s10519-025-10249-7)
Supplement: Supplementary file 1 — Supplementary Material 1 [file 10519_2025_10249_MOESM1_ESM.pdf]

Supplementary Table 1. Descriptive statistics of mental health and personality indicators.

	Men				Women				p-value of sex difference
	mean	SD	Skewness	Kurtosis	mean	SD	Skewness	Kurtosis	
Mental health									
Self-esteem	33.2	4.94	-0.73	3.01	30.4	5.54	-0.47	3.11	<0.0001
Depression	13.6	4.17	1.77	8.32	15.3	4.97	1.40	5.59	<0.0001
Alexithymia	29.7	9.42	-0.05	2.80	27.8	10.29	0.45	3.74	0.002
Schizotypal personality	5.2	4.32	0.96	3.55	6.1	4.42	0.76	3.09	0.001
Overall mental health problems	21.3	4.24	1.77	8.57	23.5	5.52	1.38	5.10	
Personality									
Neuroticism	1.4	0.63	0.42	2.86	1.9	0.70	0.37	2.82	<0.0001
Extraversion	2.5	0.46	-0.31	3.20	2.3	0.41	-0.27	3.12	<0.0001
Openness	2.0	0.54	0.34	2.94	2.2	0.51	0.16	2.52	<0.0001
Agreeableness	2.6	0.42	-0.22	3.55	2.7	0.47	-0.38	3.09	0.010
Conscientiousness	2.5	0.53	-0.39	3.19	2.6	0.54	-0.29	2.69	0.218
